# Rumen-Protected Betaine Improves Meat Quality in Tibetan Sheep by Influencing Intramuscular Fat Deposition, Antioxidant Capacity, and Fatty Acid Composition

**DOI:** 10.3390/metabo16090650

**Published:** 2026-09-04

**Authors:** Chengdi Shi, Wei Gao, Zhenglu Yang, Lijuan Han, Shengzhen Hou, Chao Yang, Zenghai Luo, Linsheng Gui

**Affiliations:** 1 College of Agriculture and Animal Husbandry, Qinghai University, Xining City 810016, China; d1876874980@163.com (C.S.); 18323977475@163.com (W.G.); 15109798269@163.com (Z.Y.); hlj880105@163.com (L.H.); qhdxhsz@163.com (S.H.); yangchaocas@126.com (C.Y.); 2 Qinghai Agri-Animal Husbandry Vocational College, Xining City 812100, China

**Keywords:** Tibetan sheep, longissimus dorsi muscle, intramuscular fat, meat quality, Genes, lipid metabolites

## Abstract

Background: Intramuscular fat (IMF) is a key determinant of meat sensory quality, but the effects of rumen-protected betaine (RPB) on IMF deposition in Tibetan sheep remain unclear. Methods: Sixty male, 3-month-old Tibetan lambs (Initial weight 17.72 ± 1.36 kg) were randomly assigned to a control (basal diet, *n* = 30) or RPB supplementation (basal diet supplemented with 0.08% RPB, *n* = 30) for 90 d. Results: Transcriptomic and lipidomic analyses revealed that RPB modulated glycerophospholipid metabolism-related genes (*DGKI* and *AGPAT1*) and altered lipid profiles, including PC (16:0/20:5) and PC (16:0/16:0). RPB increased T-AOC and SOD activities and decreased MDA content (*p* < 0.05). Intramuscular adipocyte area and diameter increased, while density decreased (*p* < 0.05). LC-MS/MS analysis showed that unsaturated fatty acids C14:1, C17:1, and C22:4 were significantly increased (*p* < 0.05). Muscle fiber density in the longissimus dorsi increased, whereas shear force, cooking loss, hardness, and chewiness were reduced (*p* < 0.05). *MYH1*, *MYH2*, and *MYH7* expression were upregulated by 2.05-, 1.89-, and 2.73-fold, respectively. Conclusions: In summary, dietary RPB supplementation was associated with increased IMF deposition, which may contribute to improved meat quality, potentially through modulation of glycerophospholipid metabolism.

## 1. Introduction

Betaine (trimethylglycine) was a naturally occurring methyl donor with established roles in lipid metabolism [1]. However, when betaine was fed directly to ruminants, it was extensively degraded by rumen microorganisms, with an in vivo degradation rate of approximately 45% per hour, which severely limited its bioavailability [2,3]. To overcome this limitation, rumen-protected betaine (RPB) was developed using lipid-coating technologies that shielded betaine from ruminal degradation, allowing it to pass intact through the rumen to the small intestine for absorption [4]. For ruminants, the biological significance of RPB was mainly reflected in three aspects. First, it served as a methyl donor via the betaine-homocysteine methyltransferase (BHMT) pathway, participating in the methionine cycle and promoting phosphatidylcholine synthesis, thereby influencing lipid metabolism [5]. Second, it acted as an osmoprotectant that accumulated within cells under hyperosmotic stress, protecting cellular structures from damage and maintaining normal cellular function [6]. Third, it improved the antioxidant defense system and alleviated oxidative stress-induced cellular damage [7].

Studies showed that dietary RPB supplementation effectively improved average daily gain and feed efficiency in lambs, while also enhancing thermoregulation, antioxidant status, and immune function [8]. A subsequent study by the same research group demonstrated that RPB supplementation improved nutrient digestibility and nitrogen retention in heat-stressed lambs, and promoted microbial protein synthesis [9]. Regarding meat quality, Dong et al. [10] found that RPB supplementation significantly reduced shear force and cooking loss in growing lambs, reflecting improved tenderness and water-holding capacity, while also increasing the contents of unsaturated fatty acids and flavor-related amino acids in muscle. Another study confirmed that RPB supplementation improved tenderness, water-holding capacity, and redness of lamb meat, and enriched umami-related amino acids [11]. Additionally, Li et al. [4] reported that RPB supplementation improved carcass traits in Hu sheep, reduced shear force, increased the contents of umami amino acids, essential amino acids, and total amino acids, and decreased the proportion of saturated fatty acids. However, whether high-altitude breeds would similarly benefit from RPB supplementation remained unclear. Tibetan sheep (Ovis aries) were an indigenous breed of the Qinghai–Tibet Plateau, well-adapted to hypoxic environments, and had long served as a vital source of meat, milk, and income for local herders. They were also known for their tender meat with low gamey odor [12,13].

Our preliminary experiment showed that 0.08% RPB improved growth performance in Tibetan sheep, prompting us to further investigate its impact on Intramuscular fat (IMF) deposition and meat quality. Based on the above research background, we proposed the following testable hypotheses: (1) RPB supplementation would regulate the expression of genes involved in lipid metabolism pathways in IMF. (2) RPB supplementation would alter lipid metabolites in IMF. (3) These transcriptional and metabolic changes might collectively promote IMF deposition in Tibetan sheep, thereby improving their meat quality. To test these hypotheses, the present study integrated transcriptomic and lipidomic analyses to comprehensively evaluate the effects of dietary RPB supplementation on intramuscular fat deposition and meat quality, with the aim of providing a theoretical basis for optimizing nutritional regulation strategies for this breed.

## 2. Materials and Methods

This animal experimentation protocol has been ethically reviewed and approved by the Animal Protection and Utilization Committee of Qinghai University (approval no. QUA-2020-0710, approved on 10 July 2020).

### 2.1. Experimental Animals, Diets, and Sample Collection

This study was carried out from April to July 2024 at Xiangka Farm, located in GongHe County, Hainan Tibetan Autonomous Prefecture, Qinghai Province, China (36°19′ N, 99°41′ E). The feeding trial lasted 100 days, consisting of a 10-day adaptation period followed by a 90-day formal experimental phase. Sixty healthy male Tibetan lambs (3-month-old, 17.61 ± 1.23 kg), with no significant difference in initial body weight between the two groups (*p* > 0.05), were allocated to two dietary treatments: CON (basal diet) and RPB (basal diet supplemented with 0.08% RPB). The RPB product used in this study contained 62.9% betaine (≥99% purity), 2.1% biotin, and 35% hydrogenated palm oil fatty acids as the coating material, and was produced by Jinan Taifei Animal Husbandry Technology Co., Ltd. (Jinan, China). An in vitro rumen degradation assay (Menke-style) conducted by the manufacturer indicated that the 12 h ruminal disappearance of RPB was no more than 15%, whereas unprotected betaine showed over 90% degradation under identical conditions, giving a rumen bypass rate of approximately 85%, consistent with previous reports [14]. The diet contained a 70:30 concentrate-to-forage ratio, with forage consisting of oat silage and oat hay in equal proportions (dry matter basis) [15]. The concentrate (powder form) and forage were mixed by hand and provided as a complete mixed ration (not pelleted), with feed and water supplied ad libitum. Meals were offered at 08:00 and 17:00 daily.

Tibetan sheep are gregarious animals and were therefore housed in groups. Each animal was provided with 2 m^2^ of floor space, along with an equal-sized exercise yard for free movement. Feed troughs (30 cm wide, 25 cm deep, and 4 m long) and a 3 m water trough were installed in each pen. All pens and equipment were regularly disinfected and cleaned to maintain hygienic conditions throughout the trial. The basal diet was formulated according to the Chinese standard for meat sheep (NY/T 816-2021 [16]), and its ingredient composition was summarized in Table 1. All mineral and vitamin levels in the experimental diets were confirmed to be within the safe limits established by Commission Implementing Regulation (EU) 2018/1039 [17].

At the end of the experiment, twelve lambs were randomly selected (6 per group) and transported to a commercial slaughterhouse. All procedures were performed in accordance with the ARRIVE guidelines 2.0 [18]. Longissimus dorsi (LD) and intramuscular fat (IMF) were collected from the left side between the 12th and 13th ribs. Tissue samples (approximately 3 × 3 cm) were fixed in 4% paraformaldehyde (Beijing Leagene Biotechnology Co., Ltd., Beijing, China) for histology, while the remainder was snap-frozen in liquid nitrogen for subsequent fatty acid, transcriptomic, and lipidomic analyses.

### 2.2. Antioxidant Status of IMF

The concentrations of total antioxidant capacity (T-AOC), superoxide dismutase (SOD), malondialdehyde (MDA), catalase (CAT), and glutathione peroxidase (GSH-Px) in IMF were determined using commercial ELISA kits (Shanghai Meilian Biotechnology Co., Ltd., Shanghai, China). IMF samples (1.0 g) were homogenized in 0.9 mL of PBS (0.01 M, pH 7.4), centrifuged at 2500× *g* for 20 min at 4 °C, and the supernatants were collected for analysis following the manufacturers’ instructions.

### 2.3. Morphological Observation of LD and IMF

In accordance with the method outlined by Han et al. [19], adipose tissue samples were fixed in 4% paraformaldehyde solution for 48 h, dehydrated through a graded ethanol series (75%, 85%, 95%, 100%, and 100%), cleared with xylene, and embedded in paraffin. Samples were cut into 3 μm sections, stained with hematoxylin and eosin (H&E), and examined under a light microscope. Adipocyte morphology was analyzed using Image-Pro Plus 6.0 software (Media Cybernetics, Inc., Rockville, MD, USA). Adipocyte number and muscle fiber types were analyzed within a defined unit area at 200× magnification. Six representative fields per section were selected for measurement, and the average value was calculated from multiple measurements for each sample.

### 2.4. Fatty Acid Profile of IMF

Fatty acids were extracted according to the method described by Folch et al. [20]. Briefly, tissue samples (50 mg) were homogenized with chloroform/methanol (2:1, *v*/*v*), and total lipids were extracted. Fatty acid methyl esters (FAMEs) were prepared following the acid-catalyzed transmethylation procedure described by Christie [21]. FAMEs were analyzed using an AB Sciex QTRAP 6500+ mass spectrometer coupled with an Exion LC-TMA system (AB Sciex, Framingham, MA, USA). Fatty acids were separated on a Waters ACQUITY UPLC BEH C18 column (2.1 × 100 mm, 1.7 µm) at 40 °C with mobile phases of (A) 0.1% formic acid in acetonitrile/water (1:1, *v*/*v*) and (B) 50% isopropanol in acetonitrile. Mass spectrometry was performed in negative ion mode with MRM acquisition. Fatty acids were identified by comparison with FAME mix standards (Supelco 37 Component FAME Mix, Sigma-Aldrich, Darmstadt, Germany) and quantified using internal standard calibration curves.

### 2.5. Meat Quality LD

Shear force was measured on cooked LD samples (core temperature 70 °C) using a tenderness tester at 1.0 mm/s, according to the Warner-Bratzler method [22]. Water-holding capacity was determined by the filter paper press method [23]. Thawing loss and cooking loss were measured following standard procedures [24]. Textural properties were evaluated using a texture profile analyzer according to the methods reviewed by Schreuders et al. [25].

### 2.6. Lipidomics Analysis

Lipid extraction was performed using the MTBE method [26]. LC-MS/MS analysis was conducted on a Vanquish Horizon UHPLC coupled with a Q-Exactive HF-X mass spectrometer (Thermo Fisher Scientific (Waltham, MA, USA)) as previously described [27]. Raw data were processed using LipidSearch software (version 5.0, Thermo Fisher Scientific, Waltham, MA, USA) for peak detection and identification. Data processing and bioinformatics analysis were performed on the Majorbio Cloud platform (cloud.majorbio.com, accessed on 20 July 2026). Differential metabolites were selected by VIP > 1 and *p* < 0.05. KEGG pathway enrichment analysis was performed using Fisher’s exact test with Benjamini–Hochberg correction (adjusted *p* < 0.05) [28].

### 2.7. Transcriptome Analysis

Total RNA was extracted from IMF using an RNA extraction kit (Invitrogen, Carlsbad, CA, USA) according to the manufacturer’s instructions. RNA integrity was assessed using an Agilent 2100 Bioanalyzer (Agilent Technologies, Santa Clara, CA, USA), and only samples with RIN ≥ 7.0 were used for library construction [29]. mRNA was enriched, fragmented, and reverse-transcribed into cDNA. Libraries were sequenced on an Illumina HiSeq 4000 platform (Illumina, San Diego, CA, USA) in paired-end 150 bp mode. Raw reads were filtered using fastp (Version 0.19.5) [30], and clean reads were aligned to the sheep reference genome (Ovis aries Oar_v3.1) using HISAT2 (v2.0.5) [31]. Gene-level read counts were quantified using featureCounts (v1.5.0-p3) [32]. Differential expression analysis was performed using DESeq2 (v1.20.0), with genes considered significantly differentially expressed at *p* < 0.05 and |log_2_ fold change| > 1 [33]. GO and KEGG enrichment analyses were performed using clusterProfiler (v3.8.1) with Benjamini–Hochberg correction (adjusted *p* < 0.05) [34].

### 2.8. Determination of MyHCs Gene Expression in LD and Validation of IMF Quantification by RT-qPCR

Total RNA was extracted from LD and IMF using TransZol reagent (TransGen Biotech, Beijing, China) according to the manufacturer’s protocol. cDNA was synthesized using the PrimeScript™ RT Kit (TaKaRa, Dalian, China). RT-qPCR was performed using SYBR^®^ Premix Ex Taq™ (TaKaRa, Dalian, China) on an Archimed R6 Real-Time PCR System (RocGene, Beijing, China). Thermal cycling conditions were 95 °C for 30 s, followed by 40 cycles of 95 °C for 10 s and 60 °C for 30 s. Melting curve analysis was performed to confirm specificity. Primers for *MYH1* (*MyHC-IIx*), *MYH2* (*MyHC-IIa*), *MYH4* (*MyHC-IIb*), and *MYH7* (*MyHC-I*) are listed in Supplementary Table S1. Relative expression was calculated using the 2^−ΔΔCt^ method, with *GAPDH* as the reference gene [35].

### 2.9. Statistical Analysis

Normality of the data was assessed using the Shapiro–Wilk test [36], and homogeneity of variances was evaluated using Levene’s test [37]. For variables with equal variances (Levene’s test *p* > 0.05), the *t*-test results under the “equal variances assumed” assumption were used. For variables with unequal variances (Levene’s test *p* < 0.05), the results under the “equal variances not assumed” assumption were used instead. Two-group comparisons were performed using the independent samples *t*-test. Data are presented as mean ± SEM, with *p* < 0.05 considered statistically significant. All statistical analyses were conducted using SPSS software (version 26.0, IBM Corp., Armonk, NY, USA) [38]. Pearson correlation analysis was performed to assess relationships among gene expression, lipid metabolites, and phenotypic traits, based on six independent biological replicates per group (*n* = 6 per group). Following common practice in multi-omics correlation studies, only correlations with |r| ≥ 0.6 and *p* < 0.05 were retained for further analysis [39]. Correlation and integrative analyses were performed in R (v4.4.1) using the corrplot package [40].

## 3. Results and Analysis

### 3.1. Antioxidant Parameters of IMF in Tibetan Sheep

Table 2 summarizes the antioxidant parameters measured in IMF. The RPB group had significantly higher T-AOC and SOD levels than the CON group (*p* < 0.05), while MDA levels were significantly lower (*p* < 0.05).

### 3.2. Morphological Observations of IMF in Tibetan Sheep

**Figure 1:**
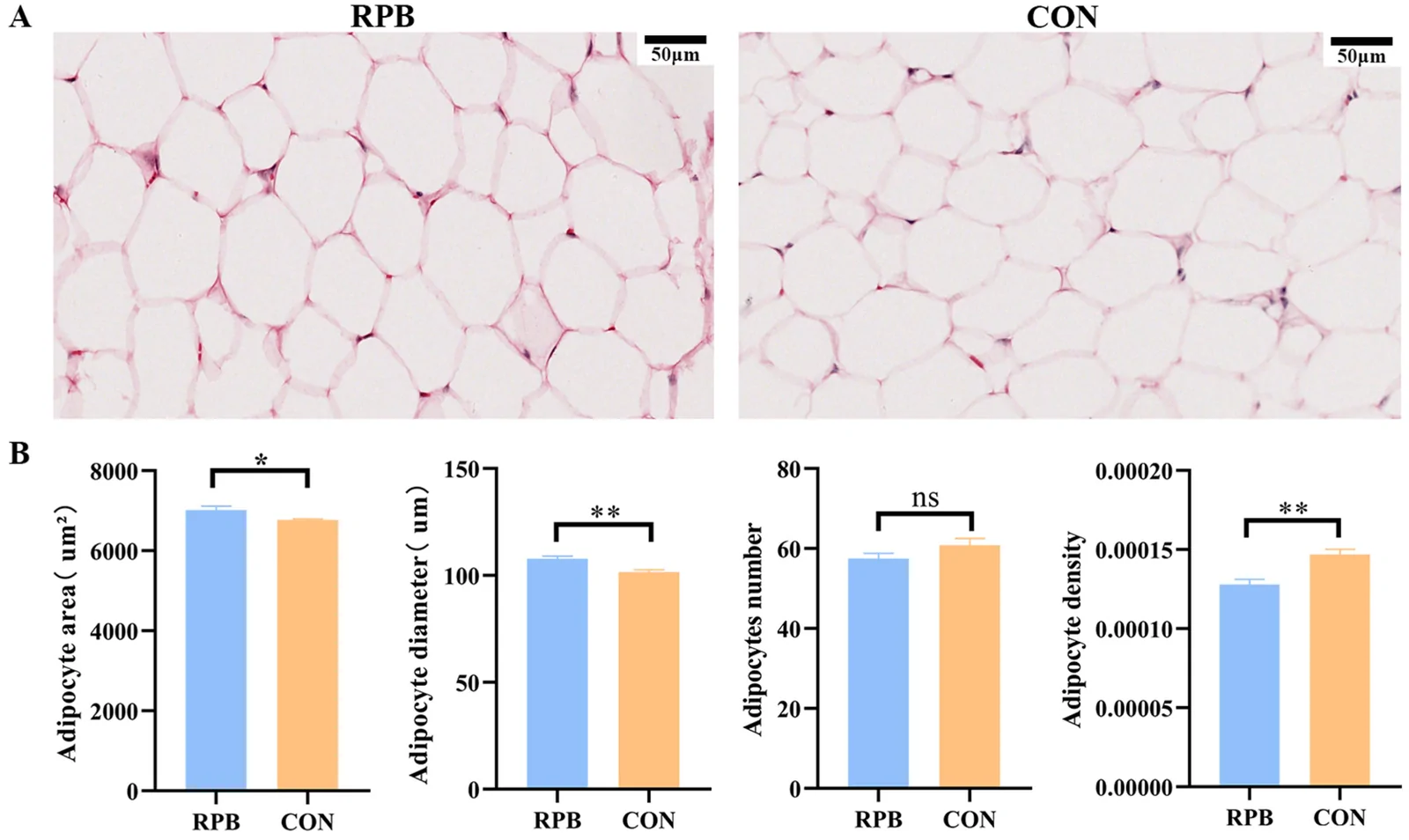
Effect of RPB on the morphology of IMF in Tibetan sheep. (**A**) Histological images of intramuscular adipose tissue with H&E staining. (**B**) Statistics of intramuscular adipocyte area, diameter, number and density. Data were presented as mean ± SEM (*n* = 6). Significance levels: * *p* < 0.05, ** *p* ≤ 0.01, ns *p* > 0.05.

H&E-stained sections of IMF tissue from both groups are shown in Figure 1. The area, diameter, number, and density of IMF cells were measured (Figure 1). The RPB group showed significantly greater IMF cell area and diameter, as well as higher cell density, compared with the CON group (*p* < 0.05), while cell number showed no significant difference between the two groups (*p* > 0.05).

### 3.3. Fatty Acid Analysis of IMF in Tibetan Sheep

A total of 49 fatty acids were detected in the IMF of Tibetan sheep (Table 3), with most fatty acids showing higher concentrations in the RPB group than in the CON group. Among these, the unsaturated fatty acids C14:1, C17:1 and C22:4 showed significant differences between the two groups (*p* < 0.05). The remaining fatty acid data are presented in Supplementary Table S3.

**Table 3:** The Effect of RPB Supplementation on Fatty Acids in IMF (ng/g).

Items	RPB	CON	*p*-Value
Unsaturated Fatty Acid			
C14:1	1836.33 ± 254.17	1091.66 ± 88.15	0.009
C17:1	4630.89 ± 587.30	2857.38 ± 620.33	0.023
C22:4	1148.51 ± 44.82	925.94 ± 117.59	0.038

Note: The statistical significance of the data was compared with Student’s *t*-test. Data were presented as mean ± SEM. The table lists three unsaturated fatty acids that showed significant differences between the two groups (*p* < 0.05). The remaining fatty acid data are presented in Supplementary Table S3.

### 3.4. Morphology of the LD Muscle, Meat Quality, and MyHCs Gene Expression Analysis

**Figure 2:**
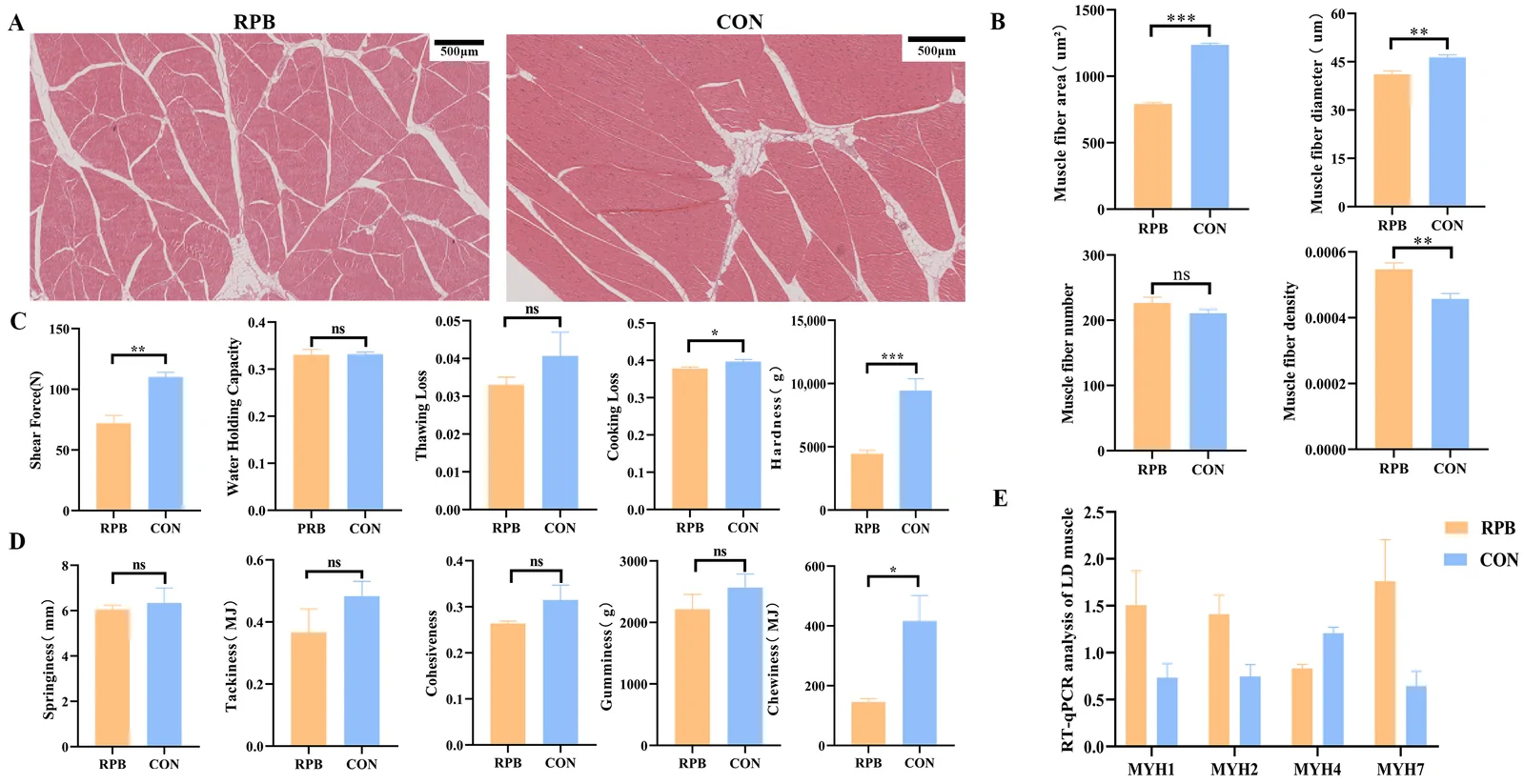
Effect of RPB on the morphology of LD tissue in Tibetan sheep. (**A**) Histological images of LD muscle tissue with H&E staining. (**B**) Statistics of myofiber area, diameter, number and density in LD muscle tissue. (**C**) Meat quality analysis of the LD muscle (Shear Force, Water Holding Capacity, Thawing Loss, Cooking Loss, Hardness. (**D**) Analysis of Meat Texture Profile Data (Springiness, Tackiness, Cohesiveness, Gumminess, Chewiness). * *p* < 0.05, ** *p* ≤ 0.01, *** *p* ≤ 0.001, ns *p* > 0.05. (**E**) Expression levels of *MyHCs* isoform genes in the two groups: *MYH1* (*MyHC-IIx*), *MYH2* (*MyHC-IIa*), *MYH4* (*MyHC-IIb*), *MYH7* (*MyHC-I*).

In this study, H&E-stained sections revealed a “marbled” distribution of fat within the LD tissue of the RPB group (Figure 2). Analysis of muscle fiber parameters (Figure 2) indicated that the muscle fiber area, diameter, and density in the LD of the RPB group were significantly altered compared with the CON group (*p* < 0.05). The RPB group exhibited significantly lower shear force and cooking loss than the CON group (*p* < 0.05, Figure 2). (*p* < 0.05). Texture profile analysis revealed that hardness and chewiness in the RPB group were significantly decreased (*p* < 0.05), while no significant differences were observed between the two groups in terms of springiness, adhesiveness, or cohesiveness (*p* > 0.05) (Figure 2). Analysis of *MyHCs* gene expression (Figure 2) revealed that, compared with the CON group, *MYH1*, *MYH2* and *MYH7* were upregulated by 2.05-, 1.89- and 2.73-fold, while *MYH4* was downregulated by 0.69-fold.

### 3.5. Lipidomics Analysis of Intramuscular Fat in Tibetan Sheep

#### 3.5.1. Identification of Lipid Compounds

**Figure 3:**
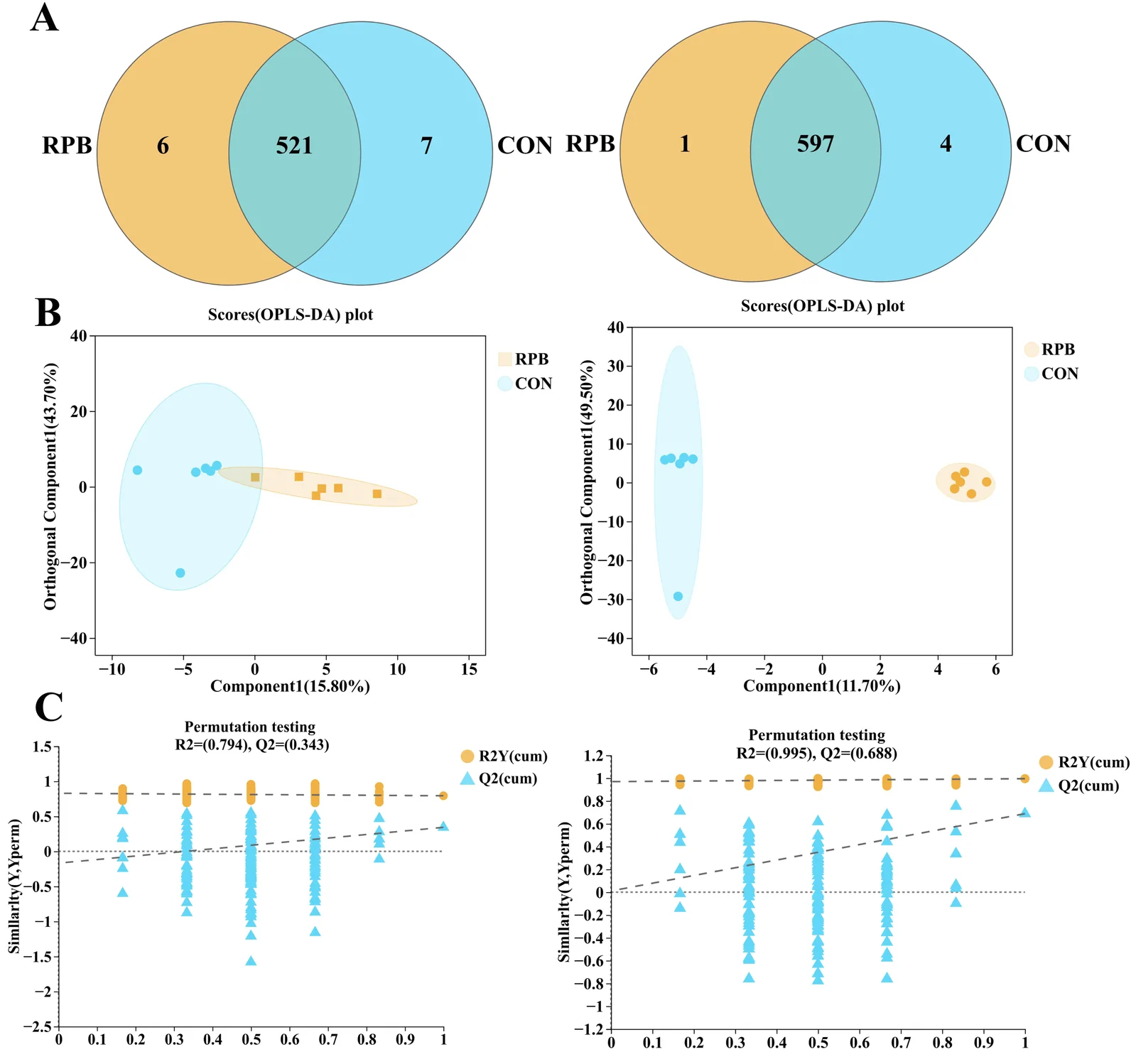
Lipidomic Analysis of IMF in Tibetan Sheep. (**A**) Venn diagrams showing the distribution of lipid metabolites detected in positive and negative ion modes for each group. (**B**) Orthogonal partial least squares discriminant analysis (OPLS-DA) score plots showing the separation of metabolic profiles between the CON and RPB groups in positive and negative ion modes. (**C**) Validation plots of the OPLS-DA models in positive and negative ion modes based on 200 permutation tests.

Venn diagram analysis revealed that, in negative ion mode, 521 lipid metabolites were shared between the two groups, with 6 uniquely expressed in the RPB group and 7 uniquely expressed in the CON group. In positive ion mode, 597 metabolites were shared between the two groups, with 1 uniquely expressed in the RPB group and 4 uniquely expressed in the CON group (Figure 3). OPLS-DA revealed a clear separation between the two groups in both positive and negative ion modes (Figure 3). Model validation results showed that both models had acceptable predictive ability, with R^2^ = 0.794 and Q^2^ = 0.343 in positive ion mode, and R^2^ = 0.995 and Q^2^ = 0.688 in negative ion mode (Figure 3).

#### 3.5.2. Differential Analysis of Lipid Compounds

**Figure 4:**
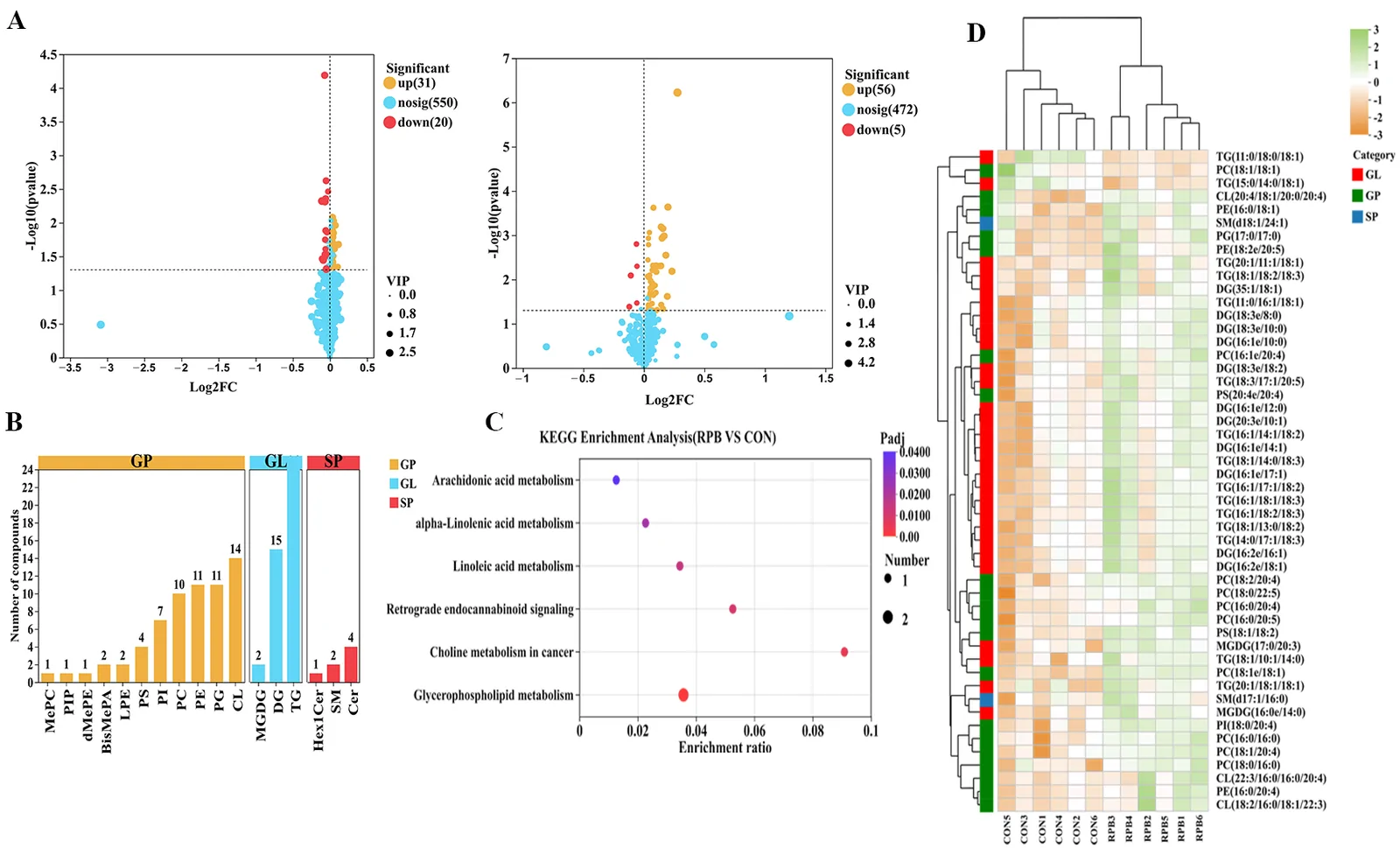
Analysis of Metabolites Associated with Differences in IMF in Tibetan Sheep. (**A**) Volcano plot: The *x*-axis represents the fold change in metabolite expression between the two groups, expressed as log_2_FC, and the *y*-axis represents the statistical significance of the differences in metabolite expression levels. Each point in the plot represents a specific metabolite, with the point size indicating the VIP value. (**B**) Legend: Different colors represent the major categories in each bar—Glycerolipids (GL), Glycerophospholipids (GP), and Sphingolipids (SP). The *x*-axis lists the names of lipids identified within each subcategory, and the *y*-axis displays the number of lipids in each subcategory. (**C**) KEGG pathway enrichment analysis: The Benjamini–Hochberg method was applied for multiple-testing correction, and pathways with adjusted *p* < 0.05 were considered significantly enriched. (**D**) Clustering heatmap: Each column in the figure represents a sample, each row represents a metabolite, and the colors indicate the relative expression level of the metabolite in the samples of that group.

Among all detected lipids, a total of 112 significantly expressed lipid metabolites were identified (VIP > 1, *p* < 0.05), which were primarily classified into three major categories: glycerophospholipids (GP), glycerolipids (GL), and sphingolipids (SP) (Figure 4). The volcano plot (Figure 4) revealed that, in positive ion mode, 31 lipid metabolites were upregulated and 20 were downregulated. In negative ion mode, 56 were upregulated, and 5 were downregulated. Hierarchical clustering of the top 50 DEMs revealed two distinct expression clusters between the CON and RPB groups (Figure 4). KEGG enrichment analysis was conducted on the differential lipid metabolites (BH, *p* < 0.05) (Figure 4). The results indicated that, among the top 20 pathways, the glycerophospholipid metabolism pathway was significantly associated with IMF deposition. Based on the results of the KEGG pathway analysis, five differentially expressed metabolites associated with IMF deposition were ultimately identified. These include the upregulated PC (16:0/20:5), PC (16:0/16:0), and PC (18:0/16:0), as well as the downregulated PC (18:1/18:1) and PG (16:0/16:0).

### 3.6. Transcriptomics Analysis of Intramuscular Fat in Tibetan Sheep

A total of approximately 52.10 million raw reads were generated from transcriptome sequencing of the 12 IMF samples (Table S2). After stringent quality control filtering, approximately 50.34 million high-quality clean reads were obtained, accounting for an average of 96.61% of the raw data. The clean data exhibited high base quality, with average Q20 and Q30 values of 97.08% and 91.88%, respectively, and an average GC content of 51.74%. These metrics confirmed the high quality and reliability of the sequencing data.

#### 3.6.1. Analysis of IMF Gene Expression

**Figure 5:**
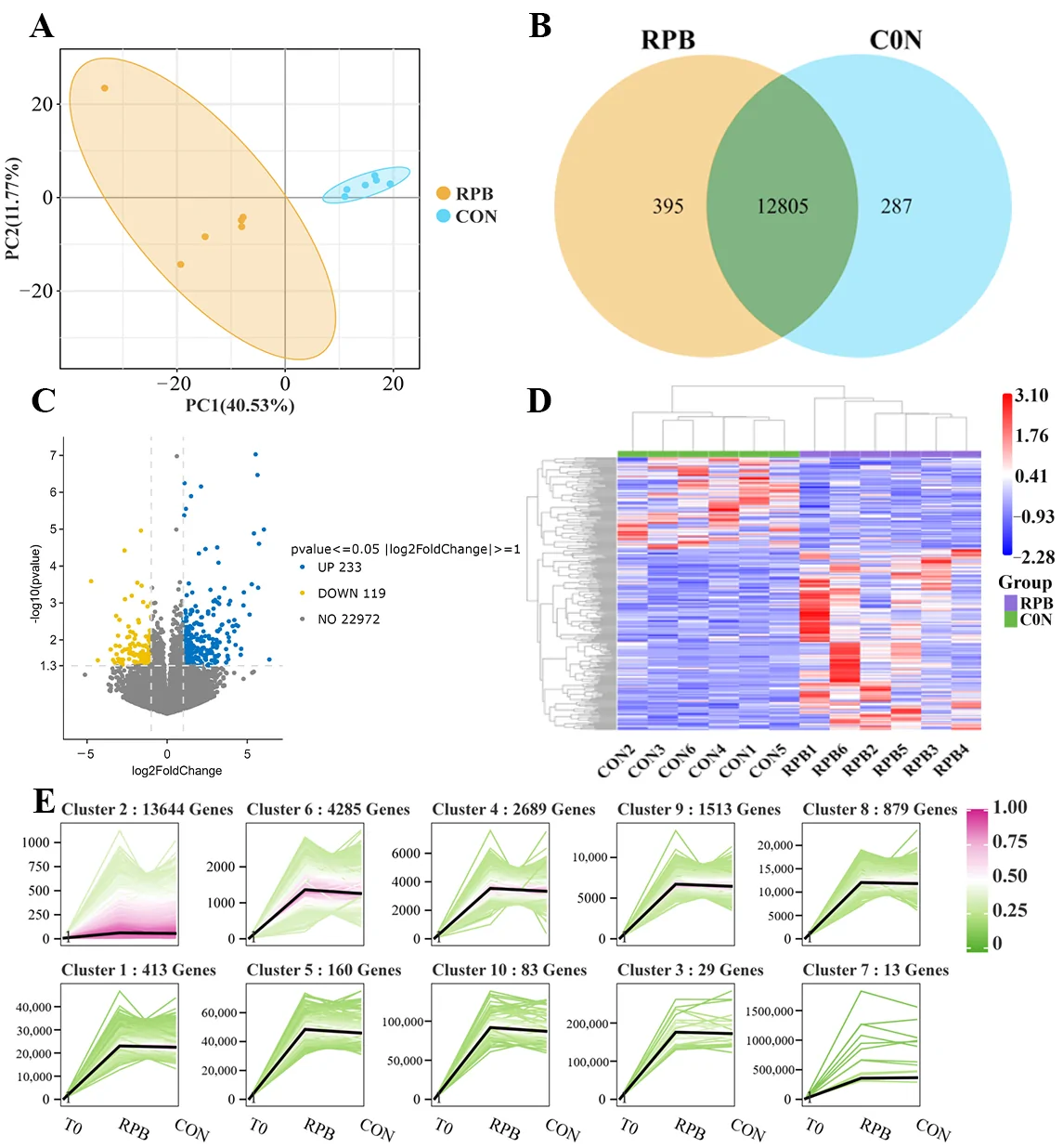
Transcriptomic Analysis of IMF in Tibetan Sheep. (**A**) Principal component analysis (PCA) plot. (**B**) Venn diagram showing the overlap of expressed genes between the CON and RPB groups. (**C**) Volcano plot showing the distribution of DEGs between the RPB and CON groups. The horizontal axis represents the fold change in gene expression between the two groups (log_2_FC), and the vertical axis represents the statistical significance of expression differences −log_10_(*p*-value), with higher values indicating more significant differences. Each dot represents a specific gene: blue dots represent upregulated genes, yellow dots represent downregulated genes, and gray dots represent genes with no significant difference. (**D**) Clustering heatmap of differentially expressed gene correlations; colors from blue to red indicate low to high relative expression. Hierarchical clustering was applied to both rows and columns. (**E**) Trend analysis (Series Test of Cluster) was performed to cluster gene expression patterns based on the characteristics of multiple consecutive samples.

Mfuzz cluster analysis classified 30,942 genes into distinct expression clusters, with clusters 2, 6, 4, and 9 representing the major patterns (Figure 5). A Venn diagram was constructed to visualize the overlap of expressed genes between the CON and RPB groups. Among the 13,487 genes included in this analysis, 12,805 genes were co-expressed in both groups, while 395 genes were uniquely expressed in the RPB group and 287 genes were uniquely expressed in the CON group (Figure 5). Principal component analysis (PCA) revealed a clear separation between the CON and RPB groups (Figure 5). Hierarchical clustering analysis of the samples from both groups showed distinct expression patterns, with colors ranging from blue to red representing low to high gene expression levels (Figure 5). Based on the criteria of |log_2_ fold change| > 1 and *p* < 0.05, a total of 352 DEGs were identified between the two groups, of which 233 were upregulated and 119 were downregulated (Figure 5).

#### 3.6.2. Enrichment Analysis of DEGs in IMF

**Figure 6:**
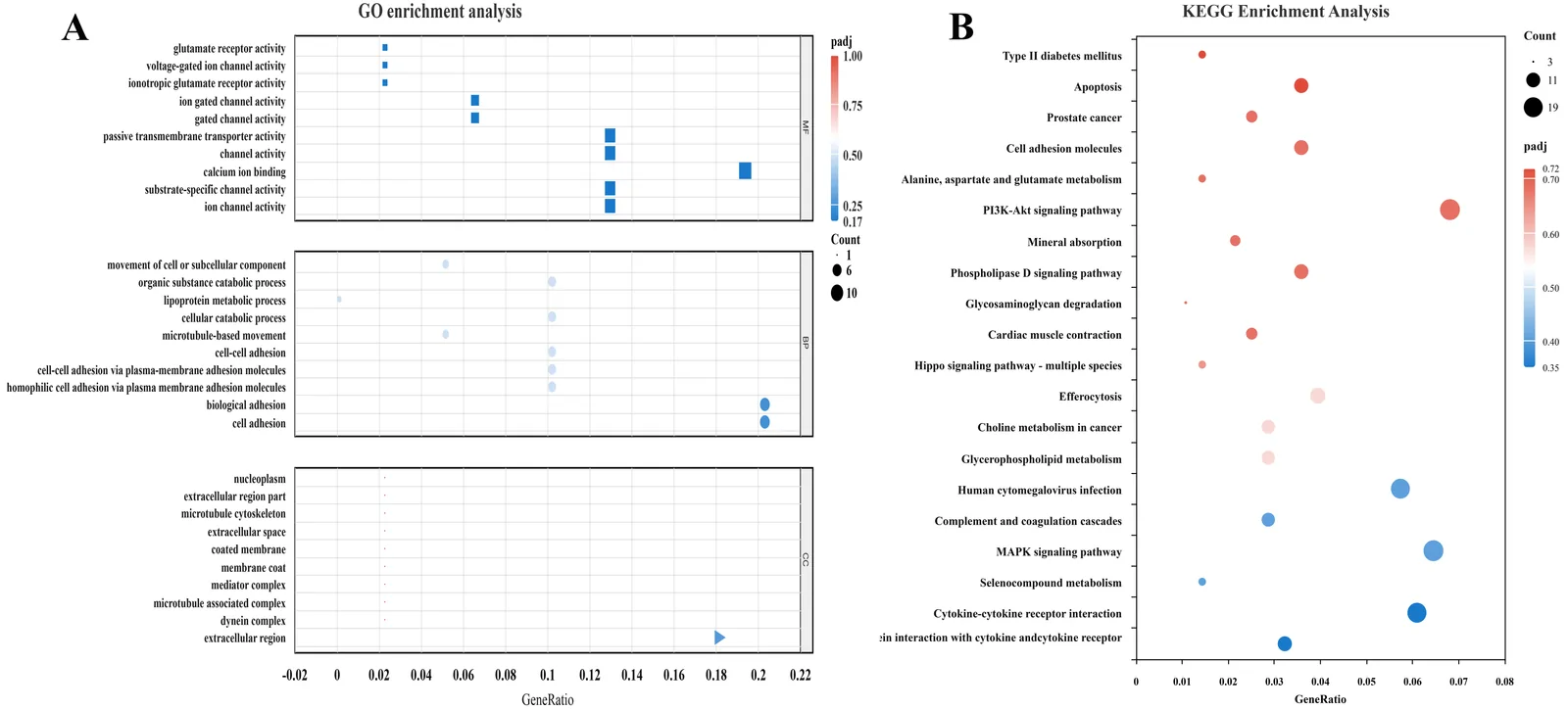
Analysis of Gene Enrichment in IMF of Tibetan Sheep. (**A**) GO enrichment analysis of DEGs: Biological Process (BP), Cellular Component (CC), Molecular Function (MF). Squares represent MF. circles represent BP. triangles represent CC. (**B**) KEGG enrichment analysis of DEGs. GO and KEGG enrichment analyses, *p*-values were adjusted using the Benjamini–Hochberg method, and significantly enriched terms were identified at *padj* < 0.05. The color scale represents the *p*-value, and the bubble size represents the number of genes enriched in each pathway.

To investigate the biological functions and associations of RPB on IMF, GO and KEGG enrichment analyses were performed on the identified DEGs, with the Benjamini–Hochberg method applied for multiple-testing correction. A total of 527 GO terms were significantly enriched (BH, *p* < 0.05), encompassing three main functional categories: biological process (BP, 260 terms), cellular component (CC, 48 terms) and molecular function (MF, 219 terms) (Figure 6). In the KEGG enrichment analysis (Figure 6), among the top 20 pathways, three pathways were identified as being significantly enriched (BH, *p* < 0.05) and associated with lipid metabolism: Cytokine-cytokine receptor interaction, MAPK signaling pathway, and Glycerophospholipid metabolism. Based on these results, 14 genes across these three pathways were selected for further analysis: in Cytokine-cytokine receptor interaction, upregulated *IL34* and *EDA2R*, downregulated *TNF*, *IL1R1* and *IL18R1*. In the MAPK signaling pathway, upregulated *NFATC1*, *MYC* and *PLA2G4A*, downregulated *GADD45A* and *DUSP4*. In glycerophospholipid metabolism, *DGKI* and *AGPAT1* were upregulated, whereas *PLA2G15* and *AGPAT4* were downregulated.

#### 3.6.3. RT-qPCR Validation of DEGs

**Figure 7:**
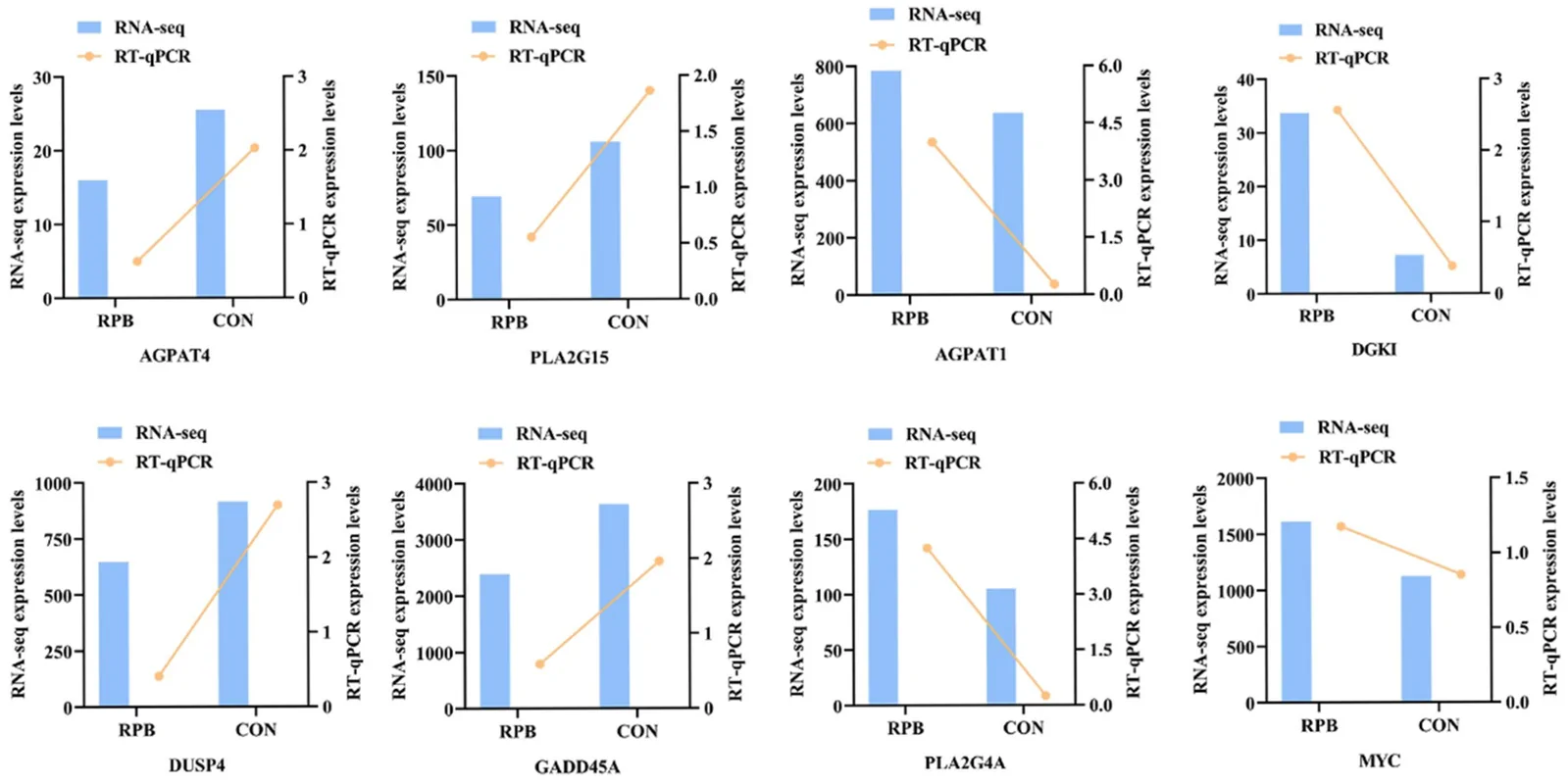
RT-qPCR Differential Gene Expression Trends. RNA-seq represents IMF transcriptomic data (shown as bar charts in the figure). RT-qPCR represents quantitative analysis data for IMF (shown as line segments in the figure).

To validate the reliability of the RNA-seq data, eight DEGs involved in three key KEGG pathways were selected for RT-qPCR analysis (Table S4). The expression trends of all eight genes were consistent with the RNA-seq results (Figure 7). Specifically, the expression levels of *AGPAT1*, *DGKI*, *MYC*, and *PLA2G4A* in the RPB group were significantly higher than those in the CON group, with fold changes of 14.85, 7.14, 1.37 and 16.38, respectively (*p* < 0.05). In contrast, the expression levels of *AGPAT4*, *DUSP4*, *GADD45A*, and *PLA2G15* in the RPB group were significantly lower than those in the CON group, with fold changes of −4.06, −7.10, −2.32 and −3.34, respectively (*p* < 0.05). These results confirmed the reliability of the RNA-seq data (Table S5).

### 3.7. Transcriptomics, Lipidomics, and Association Analysis of Phenotypic Data

**Figure 8:**
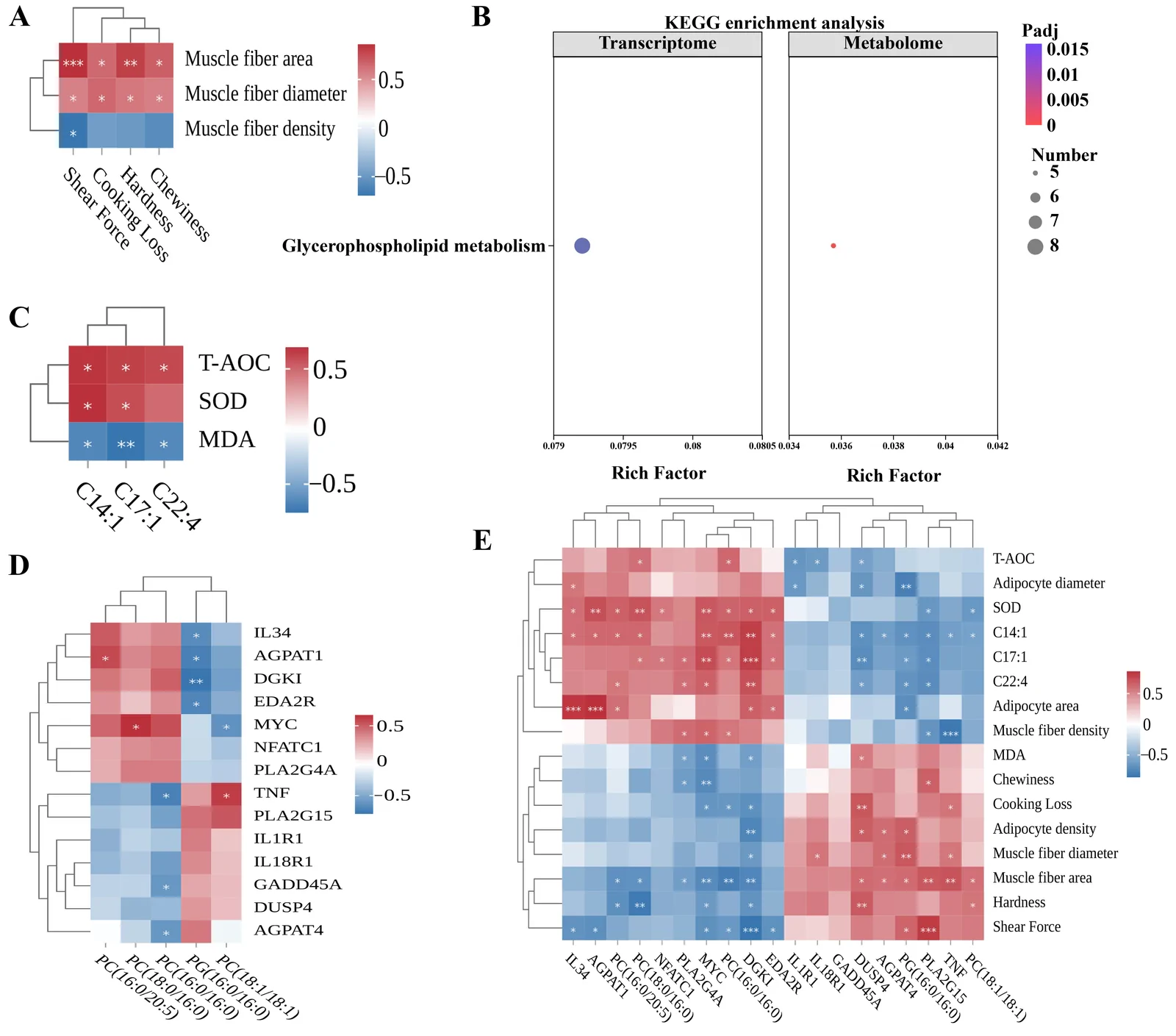
Correlation Analysis of Transcriptomic, Lipidomic, and Phenotypic Data. In the correlation analysis, |r| ≥ 0.6 (strong correlation) and *p* < 0.05 (statistically significant) were shown. Not reaching the threshold for strong correlation, a statistically significant weak-to-moderate correlation was observed between X and Y (|r| < 0.6), * 0.01 < *p* < 0.05. ** 0.001 < *p* < 0.01. *** *p* ≤ 0.001. (**A**) Correlation analysis between myofiber histological characteristics and meat quality of LD. (**B**) Integrated transcriptomic and lipidomic analysis: KEGG-enriched pathways. (The Benjamini–Hochberg method was applied to control the false discovery rate (FDR), and significantly enriched terms were identified at adjusted *p* < 0.05). (**C**) Correlation analysis between antioxidant indicators and fatty acids. (**D**) Correlation analysis between DEGs and DEMs. (**E**) Correlation analysis between DEGs and DEMs with phenotypic data.

In the integrative analysis of transcriptomic and lipidomic data, the glycerophospholipid metabolism pathway was identified as a commonly enriched pathway between the two omics datasets (*p* < 0.05, Figure 8). Based on this, the DEGs involved in the cytokine-cytokine receptor interaction pathway and the MAPK signaling pathway from the transcriptomic data, along with the candidate differentially expressed genes and metabolites in the glycerophospholipid metabolism pathway identified through the integrated analysis, were selected for further correlation analysis.

Pearson correlation analysis (|r| ≥ 0.6, *p* < 0.05) revealed that T-AOC was significantly positively correlated with C14:1 (r = 0.69, *p* < 0.05) and C17:1 (r = 0.65, *p* < 0.05). Similarly, SOD showed a significant positive correlation with C14:1 (r = 0.70, *p* < 0.05) and C17:1 (r = 0.60, *p* < 0.05)., while MDA was significantly negatively correlated with C14:1 (r = −0.64, *p* < 0.05), C22:4 (r = −0.64, *p* < 0.05) and C17:1 (r = −0.73, *p* < 0.01), (Figure 8). The area of LD muscle fibers was significantly positively correlated with shear force (r = 0.88, *p* < 0.001), hardness (r = 0.82, *p* < 0.01), cooking loss (r = 0.67, *p* < 0.05) and chewiness (r = 0.71, *p* < 0.05). The diameter of LD muscle fibers was significantly positively correlated with cooking loss (r = 0.67, *p* < 0.05) and hardness (r = 0.61, *p* < 0.05). In contrast, myofiber density exhibited a significant negative correlation with shear force (r = −0.68, *p* < 0.05) (Figure 8). Among the DEGs, *TNF* was significantly negatively correlated with PC (16:0/16:0) (r = −0.69, *p* < 0.05), and *MYC* was significantly positively correlated with PC (18:0/16:0) (r = 0.67, *p* < 0.05) (Figure 8). Additionally, the differentially expressed gene *IL-34* showed significant positive correlations with adipocyte area (r = 0.85, *p* < 0.001) and diameter (r = 0.60, *p* < 0.05), while *DGKI* exhibited significant positive correlations with the unsaturated fatty acids C17:1 (r = 0.82, *p* < 0.001), C14:1 (r = 0.82, *p* < 0.01) and C22:4 (r = 0.75, *p* < 0.01) (Figure 8). Correlation results were displayed in Figure 8, with detailed data shown in Table S6.

## 4. Discussion

In the process of IMF deposition and meat quality formation in Tibetan sheep, antioxidant capacity serves as a critical indicator for evaluating oxidative stress status and its impact on lipid metabolism [41]. Higher T-AOC contributed to maintaining the oxidative stability of IMF and reducing the risk of lipid peroxidation, thereby protecting the integrity of muscle cell membranes and preserving meat flavor components [42]. SOD, as a core enzyme in the antioxidant enzyme system, was primarily responsible for catalyzing the conversion of harmful superoxide anions (O_2_^−^) into hydrogen peroxide (H_2_O_2_), thereby terminating the propagation of oxidative chain reactions. Increased SOD activity in muscle tissue was often positively correlated with higher contents of polyunsaturated fatty acids (PUFAs) in IMF, suggesting that SOD may facilitate the deposition of high-quality fat in muscle by protecting PUFAs from oxidative damage [43]. MDA, as a terminal product of lipid peroxidation, served as a sensitive indicator reflecting the extent of oxidative damage [44]. Elevated MDA content directly suggested exacerbated lipid peroxidation in muscle tissue. Studies showed that MDA content was positively correlated with certain saturated fatty acids (e.g., C12:0 and C13:0) in IMF, while negatively correlated with PUFA content [45]. In the present study, the RPB group exhibited elevated T-AOC and SOD levels, which were positively correlated with the contents of unsaturated fatty acids C14:1, C17:1, and C22:4, while showing negative correlations with MDA. These findings were consistent with the previously established conclusions [46]. Regarding the mechanism by which RPB improved antioxidant status, previous studies have demonstrated that betaine itself possesses little direct free radical scavenging activity. Instead, its antioxidant effects are primarily mediated through indirect pathways [47]. Betaine can enhance non-enzymatic antioxidant defenses via the methionine-homocysteine cycle [48]. More importantly, betaine has been shown to directly activate the *Nrf2-Keap1-ARE* pathway by increasing the methylation level of the *Keap1* DNA promoter, thereby improving the expression of antioxidant enzymes [47]. Additionally, betaine supplementation has been reported to increase the activities of SOD and GSH-Px in sheep [49]. These findings suggest that the elevated T-AOC and SOD levels observed in the RPB group in the present study may be attributed to betaine’s indirect activation of intracellular antioxidant signaling pathways, particularly the *Nrf2*-mediated pathway, rather than its direct free radical scavenging capacity.

Previous studies unequivocally confirmed that RPB could upregulate NADPH levels and activate FTO gene expression, thereby promoting m^6^A demethylation and ultimately driving mitotic clonal expansion of intramuscular adipocytes. This mechanism may have contributed to the increase in intramuscular adipocyte area and diameter [50]. Other studies indicated that RPB could also regulate carbohydrate metabolism by inhibiting the glycolytic pathway, thereby redirecting more glucose toward triglyceride synthesis. The significant increase in IMF deposition observed in RPB-supplemented groups was directly associated with enlarged adipocyte diameter [51]. In the present study, intramuscular adipocyte area and diameter significantly increased, while adipocyte density significantly decreased. Consistent with the aforementioned literature, these findings suggested that RPB supplementation might influence IMF deposition by modulating adipocyte morphology.

Morphological observation of the LD further revealed that the RPB group exhibited more pronounced and uniformly distributed marbling. In the present experiment, the RPB group exhibited decreased myofiber area and diameter, along with increased myofiber density. Studies have demonstrated that muscles with larger myofiber diameter or cross-sectional area tended to exhibit higher shear force upon cooking, which was primarily attributed to more intense structural contraction and greater water loss during heating, leading to increased toughness, higher cooking loss, and reduced chewiness [52]. Conversely, higher myofiber density and finer myofibers per unit area were associated with lower shear force and enhanced meat tenderness [53]. Furthermore, adhesiveness, cohesiveness, and springiness remained at relatively high levels, resulting in meat that was springy but not hard, soft but not mushy, and juicy but not greasy [54]. The relationship between myofiber characteristics and meat quality has been reported: myofiber diameter was positively correlated with shear force, whereas myofiber density was negatively correlated with shear force [54]. In the present study, shear force showed a negative correlation with myofiber density and positive correlations with myofiber area and diameter, which were consistent with previous findings. In the present study, RPB supplementation significantly upregulated the expression of *MYH1* (*MyHC-IIx*), MYH2 (*MyHC-IIa*), and *MYH7* (*MyHC-I*), while downregulating *MYH4* (*MyHC-IIb*), suggesting a potential shift in muscle fiber type from fast-glycolytic toward slow-twitch and fast-oxidative fibers [55]. This finding may be of significance for understanding the molecular basis of meat quality improvement. The shift toward a more oxidative fiber phenotype could be metabolically favorable for meat quality, as slow-twitch and fast-oxidative fibers possess higher oxidative capacity and greater mitochondrial content, which may help maintain higher postmortem pH values, enhance water-holding capacity, and improve tenderness [56]. In contrast, fast-glycolytic fibers predominantly rely on glycogen metabolism, and their excessive proportion may lead to accelerated postmortem lactate accumulation and rapid pH decline, which could ultimately result in meat toughening [57]. Previous studies have suggested that muscles with higher proportions of slow-twitch *MyHC-I* and fast-oxidative *MyHC-IIa* and *MyHC-IIx* fibers, coupled with a lower proportion of fast-glycolytic *MyHC-IIb* fibers, are associated with superior meat quality [58]. In the present study, the results were generally consistent with these findings, suggesting that RPB supplementation, potentially through the synergistic effects of both structural tissue organization and fat deposition, may have promoted increased IMF content and ultimately contributed to enhanced overall eating quality of the meat, manifested as improved tenderness and the development of marbling.

The increase in C14:1 content was associated with the upregulated expression of genes such as fatty acid synthase and *SCD* [59]. In pork and beef, elevated C14:1 levels were often accompanied by a significant increase in IMF content, potentially attributable to its involvement in adipocyte differentiation and its role in promoting the conversion of preadipocytes into mature adipocytes [60,61]. C17:1, as a less common monounsaturated fatty acid, exhibited a significant increase in content that was also considered a potential marker for enhanced IMF deposition [62]. Although the specific metabolic pathway of C17:1 remained unclear, its upward trend during IMF accumulation, like that of C14:1, suggested its possible participation in the biosynthesis or elongation of long-chain fatty acids [63]. Furthermore, in animal models with myostatin (*MSTN*) gene knockout, a coexistence of muscle growth and increased IMF was observed. Studies indicated that *MSTN* gene deletion significantly elevated C22:4 levels in muscle tissue, which might have been related to alterations in IMF metabolism [64].

In transcriptomics, within the Cytokine-cytokine receptor interaction pathway, *TNF* [65], *IL1R1* [66] and *IL18R1* [67]—all pro-inflammatory cytokines—were downregulated, suggesting a possible suppression of inflammation. *EDA2R* was associated with activation of the MAPK pathway [68]. *IL-34*, a novel cytokine, was shown to significantly increase *ERK1/2* phosphorylation levels, thereby activating MAPK signaling and its expression was upregulated during adipogenesis [69]. Correlation analysis revealed that *IL-34* levels were significantly positively correlated with both adipocyte area and diameter. This result suggested that *IL-34* might be associated with adipocyte enlargement. Meanwhile, we observed a reduction in inflammation-related indicators, and it was speculated that a microenvironment with reduced inflammation might favor adipogenesis, thereby supporting the morphological enlargement of adipocytes. Upon activation of the MAPK pathway, *NFATC1* was upregulated [70]. As a calcium-regulated calcineurin-dependent transcription factor, *NFATC1* was confirmed by multiple studies as a core positive regulator of adipocyte differentiation, with its activation marking the onset of adipogenesis [71]. The pro-proliferative regulatory factor *MYC* was concurrently upregulated [72]. Increased phospholipase activity of *PLA2G4A* led to phospholipid hydrolysis and release of arachidonic acid (AA), a process critical for lipid signal transduction and lipid synthesis [73]. Conversely, the growth arrest and DNA damage-inducible factor *GADD45A* [74] was downregulated, and downregulation of *DUSP4* was shown to affect the expression of inflammatory factors such as *IL-1β* and *TNF-α* [75]. This cascade of changes suggested that cell proliferation and differentiation might be enhanced.

In the glycerophospholipid pathway, *DGKI*, the gene encoding diacylglycerol kinase iota, was upregulated. Its primary function involved the phosphorylation of diacylglycerol (DG) to generate phosphatidic acid (PA) [76]. Previous studies have reported that *DGKI* expression was associated with phenotypic traits characterized by higher intramuscular fat content, suggesting its potential involvement in fat deposition [77]. In the present study, *DGKI* showed a significant positive correlation with unsaturated fatty acids, indicating that it may serve as a potential regulatory factor associated with fat deposition. AGPAT1 was also upregulated, which was responsible for catalyzing the acylation of lysophosphatidic acid (LPA) to form PA [78]. PA was a crucial intermediate formed during the esterification of fatty acids to a glycerol backbone and was used for the synthesis of phospholipids (e.g., PC, PE) and triglycerides (TAG), providing more substrates for triglyceride synthesis [79]. In correlation analysis, *AGPAT1* expression was positively correlated with IMF area, suggesting that *AGPAT1* may be a potential regulatory factor associated with fat deposition. However, these correlations do not establish a causal relationship, and further functional studies are warranted to confirm the precise roles of *DGKI* and *AGPAT1* in regulating IMF deposition. *PLA2G15*, a key lysosomal BMP (bis[monoacylglycerol]phosphate) hydrolase, was downregulated. Its decreased expression, leading to increased intracellular BMP, was associated with reduced aberrant cholesterol accumulation, contributing to the maintenance of cell membrane phospholipid balance and preventing excessive intracellular lipid deposition [80]. *AGPAT4*, also involved in converting LPA to PA, was downregulated, which might have reduced PA production. However, combined with morphological observations of the LD tissue, it appeared that *DGKI* and *AGPAT1* played dominant roles, while *AGPAT4* and *PLA2G15* could be involved in preventing excessive IMF deposition [81].

The upregulated phosphatidylcholines (PC) identified were major components of cell membranes and the outer layer of lipid droplets [82]. In muscle cells, the upregulation of PC (16:0/16:0) was interpreted as a protective response, helping cells resist oxidative stress while also providing an energy reserve [83]. Correlation analysis showed a negative correlation with *TNF*, which is consistent with the results of the aforementioned report. PC (18:0/16:0) could provide more saturated fatty acid precursors to muscle cells, promoting triglyceride (TG) synthesis and storage [84]. *MYC* [85], a transcription factor that plays a key role in cell proliferation, was significantly positively correlated with PC (18:0/16:0) in the correlation analysis, suggesting that it might be associated with triglyceride synthesis. The PC molecule PC (16:0/20:5) could increase membrane fluidity and promote lipid droplet formation and expansion [86]. Its significant positive correlation with *AGPAT1*, a key enzyme in the PA synthesis pathway whose catalytic product PA is an important precursor for various phospholipids including PC (16:0/20:5), suggested its potential involvement in lipid accumulation [87]. PG (16:0/16:0), an important component of the inner mitochondrial membrane, was downregulated. This downregulation could lead to a decreased rate of fatty acid breakdown within cells, redirecting them toward lipid deposition in muscle tissue [88]. It was significantly negatively correlated with unsaturated fatty acids, suggesting a potential influence on fatty acid degradation and IMF accumulation. Conversely, the decreased content of PC (18:1/18:1) was associated with improved lipid metabolic disorders, thereby reducing abnormal lipid accumulation [89]. This indicated that while RPB could influence IMF deposition, it might also have relatively inhibited excessive deposition, ensuring healthy and beneficial fat accumulation.

Several limitations of this study should be acknowledged. First, this study was conducted on a single breed (Tibetan sheep) at a specific growth stage, which may limit the generalizability of our findings to other breeds or age groups. Future studies incorporating multiple breeds and developmental stages are needed to validate the broader applicability of our conclusions. Second, meat quality assessments were performed at a single post-slaughter time point, without monitoring changes during aging or extended storage. Future research should include long-term post-slaughter evaluations to better understand the effects of RPB on meat quality traits. Third, the candidate genes and metabolites identified in this study were derived from transcriptomic and lipidomic analyses but lacked functional validation. While our multi-omics approach revealed correlations and potential regulatory networks, causal relationships remain to be established. Future studies employing targeted functional approaches—such as gene knockdown or overexpression in adipocyte models (e.g., *AGPAT1*, *DGKI*), or dietary intervention with specific metabolite supplementation—are warranted to confirm their roles in IMF deposition and meat quality regulation. Despite these limitations, our findings offer insights into how RPB influences IMF deposition and provide a foundation for future research to enhance meat quality in Tibetan sheep.

## 5. Conclusions

In conclusion, dietary RPB supplementation effectively promoted intramuscular fat deposition and improved meat quality in Tibetan sheep, likely through modulation of the glycerophospholipid metabolism pathway. These findings support RPB as a promising functional feed additive for precision nutrition in intensive feeding systems. However, certain limitations should be acknowledged. First, only a single dose (0.08%) was tested in this study, and dose–response studies are warranted to determine the optimal supplementation level. Future research is also needed to validate the functional roles of *AGPAT1* and *DGKI* in glycerophospholipid metabolism-mediated IMF deposition through targeted gene manipulation, and to evaluate the practical efficacy of RPB under diverse feeding conditions.

## Figures and Tables

**Table 1:** Composition and nutritional level of the diet concentrate (based on dry matter %).

Items	
Corn	48.40
Palm meal	16.00
Rapeseed meal	15.00
Wheat	9.00
Premix ^1^	4.60
Soybean meal	4.00
Salt	1.00
Limestone powder	1.00
Baking soda	1.00
Total	100.00
Nutrient levels	
Neutral Detergent Fiber (NDF)	21.18
Acid Detergent Fiber (ADF)	14.72
Crude protein	13.69
DE/(MJ·kg^−1^) ^2^	12.66
Ether extract	4.61
Calcium (Ca)	0.85
Phosphorus (P)	0.41

Note: ^1^ Each kilogram of premix provides the following nutrients: Cu, 18 mg. Fe, 66 mg. Zn, 30 mg. Mn, 48 mg. Se, 0.36 mg. I, 0.6 mg. Co, 0.24 mg. vitamin A, 24,000 IU. vitamin D, 4800 IU. vitamin E, 48 IU. ^2^ Digestible energy is a calculated value. All other values are measured values.

**Table 2:** Effects of Supplementation RPB on the Antioxidant Status of IMF in Tibetan Sheep.

Items	RPB	CON	*p*-Value
T-AOC (U/mL)	19.35 ± 1.47	17.34 ± 0.85	0.016
SOD (ng/mL)	21.89 ± 1.12	19.72 ± 1.46	0.016
CAT (pg/mL)	25.24 ± 0.63	25.40 ± 1.29	0.794
MDA (nmol/L)	19.81 ± 0.60	21.71 ± 1.68	0.026
GSH-Px (ng/mL)	21.39 ± 2.24	20.35 ± 2.28	0.443

Note: The statistical significance of the data was compared with Student’s *t*-test. Data were presented as mean ± SEM. Total antioxidant capacity (T-AOC), superoxide dismutase (SOD), malondialdehyde (MDA), Catalase (CAT) and Glutathione Peroxidase (GSH-Px).

## Data Availability

The datasets generated by this study are available through online storage platforms. Specifically, these datasets can be accessed via the NCBI SRA platform (accession number PRJNA1438630) and the OMIX platform (accession number PRJCA060228, PRJCA053120).

## Supplementary Materials

The following supporting information can be downloaded at: https://www.mdpi.com/article/10.3390/metabo16090650/s1, Table S1: Myosin Heavy Chain Primers used in RT-qPCR; Table S2: Differential Analysis of RNA Sequencing Data; Table S3: The Effect of RPB Supplementation on Fatty Acids in IMF (ng/g); Table S4: Primers used in RT-qPCR; Table S5: RT-qPCR validation of selected differentially expressed genes in the IMF; Table S6: Selected correlations among DEGs, DEMs, and phenotypic date identified in the correlation analysis.

## Abbreviations

**Item.**	**Term**
RPB	Rumen-Protected Betaine
CON	Control Group
IMF	Intramuscular Fat
LD	Longissimus Dorsi
MyHCs	Myosin Heavy Chains
T-AOC	Total Antioxidant Capacity
SOD	Superoxide Dismutase
MDA	Malondialdehyde
GSH-Px	Glutathione Peroxidase
CAT	Catalase
DEG	Differentially expressed gene
DEM	Differentially expressed metabolite
H&E	Hematoxylin and Eosin
QC	Quality Control
AA	Arachidonic Acid
BMP	Bis (Monoacylglycerol) Phosphate
PC	Phosphatidyl Choline
TG	Triglyceride
PA	Phosphatidic Acid
LPA	Lysophosphatidic Acid
