# Supplementary material for: Rumen-Protected Betaine Improves Meat Quality in Tibetan Sheep by Influencing Intramuscular Fat Deposition, Antioxidant Capacity, and Fatty Acid Composition
Source: Metabolites. 2026 Sep 4;16(9):650. doi: 10.3390/metabo16090650 (PMC13609058; doi:10.3390/metabo16090650)
Supplement: Supplementary file 1 [file metabolites-16-00650-s001.zip › metabolites-4514052-supplementary.pdf]

Table S1. Myosin Heavy Chain Primers used in RT-qPCR

Items	Primer sequence （5'-3'）	TM (℃)	Product Length
MYH1	F-GAGAGGAGCAGGAAGATCGC<br>R-TGTCCTCCATCTCTCCCTGG	58	145bp
MYH4	F-GCACCAGCGCCTAATCAATG<br>R-TCGGGATAGCTGAGACACCA	58	115bp
MYH2	F-GGTCTTCTGGGGCTCCTAGA<br>R-ACTCCCTCCTCTCCACCATC	59	127bp
MYH7	F-CACGCATAGAGGAGCTGGAG<br>R-CGCGCTTCTTGTTCATCTCG	58	165bp
GAPDH	F-ACAGTCAAGGCAGAGAACGG<br>R-CCAGCATCACCCCACTTGAT	58	98bp

Table S2. Differential Analysis of RNA Sequencing Data

Sample	Raw_reads	Raw_bases	Clean_reads	Clean_bases	Q20	Q30	GC_pct（%）
RPB1	52170842	7.83G	50241560	7.54G	97.25	92.34	51.51
RPB2	41685098	6.25G	39858140	5.98G	96.90	91.36	51.53
RPB3	42103176	6.32G	40438394	6.07G	96.98	91.7	52.01
RPB4	42129122	6.32G	40518912	6.08G	96.92	91.46	51.84
RPB5	39823526	5.97G	38353556	5.75G	96.99	91.63	51.55
RPB6	49230980	7.38G	47377710	7.11G	97.35	92.64	52.03
CON1	47267984	7.09G	45417402	6.81G	97.35	92.62	51.95
CON2	41161626	6.17G	40143572	6.02G	96.96	91.57	51.21
CON3	43092054	6.46G	42052338	6.31G	97.13	92.00	51.38
CON4	40814860	6.12G	39709022	5.96G	96.84	91.25	52.20
CON5	40242938	6.04G	39037478	5.86G	97.01	91.72	51.87
CON6	41374836	6.21G	40270040	6.04G	97.23	92.24	51.82

Table S3. The Effect of RPB Supplementation on Fatty Acids in IMF (ng/g).

Items	RPB	CON	*P*-value
**Saturated Fatty Acid**			
C8:0	95.07±24.33	81.34±13.05	0.437
C10:0	1070.24±272.22	860.62±70.63	0.266
C11:0	59.95±8.20	46.75±3.81	0.065
C12:0	4667.03±1953.24	3785.96±618.76	0.498
C13:0	178.16±59.04	114.31±8.04	0.137
C14:0	21768.097±7685.57	14960.16±827.20	0.202
C15:0	4904.91±1815.02	2729.30±30.72	0.107
C16:0	116735.88±24839.84	85287.61±16781.93	0.143
C17:0	8336.24±1519.27	6022.15±392.67	0.063
C18:0	132942.86±25904.36	120396.74±32873.51	0.631
C20:0	2714.89±94.79	3140.17±1062.67	0.528
C21:0	208.44±13.94	216.66±64.60	0.840
C22:0	633.64±64.28	822.14±128.59	0.086
C23:0	639.35±83.96	758.72±95.62	0.180
C24:0	1300.92±183.53	1480.88±148.38	0.257
**Unsaturated Fatty Acid**			
C14:1 T	368.41±142.63	204.00±68.22	0.146
C15:1	37.56±8.95	29.79±3.01	0.227
C15:1 T	91.72±7.47	62.93±26.17	0.141
C16:1	10925.47±3195.67	5861.47±328.36	0.052
C16:1 T	1442.53±1096.15	1089.83±703.93	0.664
C17:1 T	400.61±128.74	238.27±54.14	0.114
C18:1(n-12)	1872.07±670.29	1292.35±447.28	0.281
C18:1(n-9)	15465.39±4790.74	10818.07±3866.76	0.261
C18:1(n-7)	1264.53±1156.43	1420.46±882.15	0.862
C18:1(n-12) T	64183.69±14476.49	51700.71±14275.97	0.348
C18:1(n-9) T	66800.32±14263.67	52213.16±9069.27	0.209
C18:1(n-7) T	13222.11±6873.71	9214.20±3875.41	0.429
C18:2(n-6)	23005.00±8285.48	19020.71±4865.44	0.512
C18:2(n-6) T	2382.51±775.41	1658.59±363.05	0.217
C19:1(n-12) T	612.55±174.93	617.26±351.34	0.984
C18:3(n-6)	1986.77±623.91	1292.50±218.21	0.143
C20:1	886.11±52.81	857.63±385.31	0.905
C20:1 T	3539.69±547.05	2611.88±958.79	0.310
C18:3(n-3)	6615.10±1976.96	5887.99±659.97	0.578
C20:2	579.37±106.38	522.68±75.51	0.494
C20:3(n-6)	1039.45±82.95	851.01±115.60	0.083
C22:1	441.08±25.26	552.07±204.00	0.403
C22:1 T	1727.67±348.61	1541.02±578.72	0.657
C20:3(n-3)	951.98±50.29	883.29±107.18	0.372
C20:4	12568.51±1306.17	10770.76±2242.82	0.296
C22:2	54.62±7.05	49.31±13.59	0.581
C20:5	2432.83±81.78	2223.43±310.53	0.322
C22:5(n-3)	241.55±40.69	235.74±38.67	0.866
C22:5(n-6)	3511.29±268.19	2964.19±619.49	0.233
C24:1	557.73±5.96	630.84±60.79	0.171
C22:6	647.08±52.04	535.18±53.39	0.060

Table S4. Primers used in RT-qPCR

Items	Primer sequence (5'-3')	TM (℃)	Product Length
GAPDH	F-CTGACCTGCCGCCTGGAG<br>R-GTAGAAGAGTGAGTGTCGCTGTTG	60	149bp
AGPAT4	F-GTTCTTGTCTTCTTCGTGGCTTCC<br>R-CTTGCTGTCCATGTTGCCGTAG	59	96bp
PLA2G15	F-GAAGGTGTGGATGTGCGTGTC<br>R-TCCTTGCCTCGTTCGTAGCC	60	140bp
AGPAT1	F-CCTGCTGCTGCTCCTCCTG<br>R-TCCTCGCACGGCACACAC	62	136bp
DGKI	F-GTGACTTGGAGACTTGCCGTATG<br>R-TCGTGGTCCTGGTAATGAACTCTC	58	97bp
DUSP4	F-ACTCCGCCGTCATCGTCTAC<br>R-AACCTTTCATAGCCACCTTTCAGTAG	58	145bp
GADD45A	F-CGGACGAGGACGACGACAG<br>R-GGTTGCTGACACGCAGGATG	61	102bp
PLA2G4A	F-CTGGATGAAGACGAACTGGATGC<br>R-AAGGGTATGGAAGGTTAAATGTGAGG	57	133bp
MYC	F-TACCACCAGCAGCAGCAGAG<br>R-TACGACGGCGAGCAGAGC	61	128bp

Table S5. RT-qPCR validation of selected differentially expressed genes in the IMF

Items	RPB	CON	Fold Change (RPB/CON)	log_2FC	*P*-value
AGPAT4	0.50±0.06	2.03±0.27	-4.06	-2.02	0.001
PLA2G15	0.56±0.15	1.87±0.44	-3.34	-1.74	0.008
AGPAT1	4.01±1.50	0.27±0.08	+14.85	+3.89	0.049
DGKI	2.57±0.20	0.36±0.06	+7.14	+2.84	0.001
DUSP4	0.41±0.15	2.91±1.05	-7.10	-2.83	0.015
GADD45A	0.59±0.24	1.37±0.01	-2.32	-1.22	0.030
PLA2G4A	4.26±1.37	0.26±0.10	+16.38	+4.03	0.036
MYC	1.18±0.13	0.86±0.93	+1.37	+0.46	0.027

Table note: Data were presented as mean ± SEM. Relative expression levels were calculated using the $2^{-\Delta\Delta Ct}$ method and the results were expressed as the ratio of RPB to CON. To facilitate result interpretation and align with transcriptomic data reporting standards, both fold change and log_2FC were presented. Specifically: (1) Fold change (RPB/CON) directly reflected the magnitude of expression difference between the two groups—values > 1 indicated upregulation in the RPB group, values < 1 indicated downregulation, and values equal to 1 indicated no change; (2) log_2FC (log2-transformed fold change) was the metric used by DESeq2 for differential expression analysis and offered the advantage that upregulation and downregulation of the same magnitude were symmetrically distributed around zero. This dual presentation ensured consistency with the RNA-seq data analysis pipeline and facilitated direct comparison between RT-qPCR validation and transcriptomic results. Positive fold change values indicated higher expression in the RPB group (upregulation), whereas negative values indicated lower expression in the RPB group (downregulation). *P*-values were calculated using independent samples t-tests, and $P < 0.05$ was considered statistically significant.

Table S6 Selected correlations among DEGs,DEMs,and phenotypic date identified in the correlation analysis.

Var1	Var2	cor	*P*_value
T-AOC	C14:1	0.69	0.014
SOD	C14:1	0.70	0.011
MDA	C14:1	-0.64	0.024
T-AOC	C17:1	0.65	0.022
SOD	C17:1	0.60	0.037
MDA	C17:1	-0.73	0.007
MDA	C22:4	-0.64	0.026
TNF	PC (16:0/16:0)	-0.69	0.013
MYC	PC (18:0/16:0)	0.67	0.018
Muscle fiber area	Shear Force	0.88	0.000
Muscle fiber density	Shear Force	-0.68	0.015
Muscle fiber area	Cooking Loss	0.67	0.018
Muscle fiber diameter	Cooking Loss	0.67	0.016
Muscle fiber area	Hardness	0.82	0.001
Muscle fiber diameter	Hardness	0.61	0.035
Muscle fiber area	Chewiness	0.71	0.010
Adipocyte area	IL34	0.85	0.000
Adipocyte diameter	IL34	0.60	0.040
C14:1	DGKI	0.82	0.001
C17:1	DGKI	0.82	0.001
C22:4	DGKI	0.75	0.005

Note: Pearson correlation analysis ($|r| \geq 0.6$, $P < 0.05$). cor: Pearson correlation coefficient, positive and negative values indicate positive and negative correlations, respectivel.
